# Opening the black box: Understanding the editorial and peer review journey of a manuscript

**DOI:** 10.1177/02692163251406788

**Published:** 2025-12-20

**Authors:** Catherine Walshe, Debbie Ashby

**Affiliations:** 1 International Observatory on End of Life Care, Lancaster University, UK; 2 Palliative Medicine Journal Office, UK

Submitting a paper to a research journal is the culmination of months, often years, of research and writing. Yet for authors, what happens after pressing the “submit” button remains largely unseen. Peer review and editorial processes can feel opaque, protracted, or unpredictable. Understanding the steps that follow submission can help set appropriate expectations and facilitate a transparent partnership between authors, reviewers, and editors. This aim of this editorial is to provide a clear account of how manuscripts are processed at Palliative Medicine, from initial submission through to editorial decision-making. Table 1 summarises this process, with indicative timelines, and an explanation of your author view status within the online submission system.

**Table 1.** Editorial and peer review stages.

Stage	Editors’ tasks and typical timescale	Your view on the submission system
1. Initial submission and administrative checks.	Administrative checks (up to 2–4 days)	Awaiting Editorial Manager Processing
2. Editorial triage	Determining whether to send to review or not (up to 3 weeks)	Awaiting referee selection
3. Inviting reviewers	Selecting reviewers (anything from 1 week to 6+ months depending on speed of response of invited reviewers and number of rounds of invitations needed)	Awaiting referee selection
4. In peer review	Two reviewers have agreed to review (reviews not yet returned)	Awaiting referee assignment
5. Editorial decision making	Making first and subsequent decisions based on reviewer and editors’ assessment	Awaiting final decision
6. Revision and resubmission	Awaiting revised papers, sending to reviewers, making subsequent decisions.	Awaiting revision (then) Awaiting final decision

## Stage 1. Initial submission and administrative checks

Your first step is to submit via our online system. These systems are typically not managed by journals themselves but adopted by publishing houses. They are used for multiple journals, and hence we have little control over their functionality. Once your paper is submitted, we first make a series of checks to ensure that the correct files have been uploaded and that they are not corrupted or otherwise unreadable. At this point we are not evaluating the scientific quality of your manuscript but verifying that the submission is complete and (mostly) compliant with our author guidelines.

We may return your paper to you almost immediately if you have not uploaded both of our mandatory checklists, or if you upload an incomplete or unfinished version of your paper. At Palliative Medicine we are relatively relaxed about some aspects at this stage (e.g. formatting, or that it contains required statements and declarations) as these can often be addressed during the reviewing process. However, providing these aspects does help us to have clear and appropriately documented work to assess, and attention to detail is the hallmark of good research. This stage typically will happen within a few days, bear in mind that the none of those managing this stage work full time.

## Stage 2. Editorial triage

After administrative checks, the manuscript moves to the five screening editors for initial evaluation. At this stage, the editors consider whether the paper aligns with the journal’s aims and scope, contributes meaningfully to current scholarship, and meets a baseline standard of clarity and methodological soundness. This stage is sometimes referred to as “triage” or “desk review.” Around two-thirds of manuscripts are declined at this point, often because the research question is not sufficiently novel, the methodological approach does not support the conclusions or has not been sufficiently robustly conducted, or the paper does not appropriately engage with, and contribute to, the international palliative care evidence base. We have written about writing for our international audience previously.^1^

We aim to complete this stage within 3 weeks – but please be patient with us if it takes a little longer. Our screening editors all have busy jobs as clinicians and researchers in palliative care. Their screening work is typically undertaken at weekends and evenings. If we take a little longer than you would like, please do contact us, but please remember that you are not corresponding with a full-time editorial team.

## Stage 3. Inviting reviewers

For manuscripts that progress beyond initial triage, we then move on to inviting peer reviewers. Editors seek reviewers who possess relevant subject matter expertise, familiarity with the methodological approach, and the capacity to deliver balanced, high-quality feedback. We also consider diversity in reviewer backgrounds, and their perspectives may reflect different but complementary evaluative traditions. We will often invite reviewers from different countries to your own to reflect on the international transferability or understanding of your work. Authors are not permitted to suggest reviewers themselves.

Reviewers are given a 2 week period in which to accept or decline. It is not uncommon for invitations to be issued over multiple tranches to secure at least two independent expert reviews. Because reviewers volunteer their time, securing reviewers may require patience. It is increasingly common for us to have to invite 20+ reviewers before we secure two agreed reviewers. This builds delay into the systems whilst we wait for people to decline (most do not respond at all, unfortunately) before inviting the next tranche of reviewers. During this time you don’t see any change in your manuscript status so we understand that you might think we are not actively managing your paper. The online system manages this automatically – please don’t think we have forgotten a paper because you have not heard from us. Once potential reviewers have declined to review or the system auto-declines them after 2 weeks the manuscript automatically returns to the section of the submission site where we will invite more potential reviewers.

We are proud of the fact that we have never declined a paper because we could not identify reviewers. We manage this through inviting a wider range of external experts, often from a variety of disciplines. If necessary, we invite members of our editorial and editorial advisory boards to conduct reviews.

## Stage 4. In peer review

Once we have agreed reviewers, they receive access to the manuscript and supporting materials. Reviewers assess the work across several criteria: significance and originality of the research question, appropriateness and rigour of the methodology, clarity and transparency of reporting, relevance and strength of interpretation, and contribution to clinical, theoretical, or policy discussions within palliative care.^2^

We give reviewers 3 weeks to submit their review but will often extend this timescale for people to enable them to commit to a review within their busy schedules. Sometimes people who have promised to review a paper never return a review, despite personalised reminders. In which case its back to the start for us (and a further delay for your paper) whilst we source another reviewer. Many manuscripts that are ultimately accepted undergo multiple rounds of revision.

## Stage 5. Editorial decision-making

After the reviews are completed, the editor reviews the comments and forms an assessment. Reviewer recommendations are advisory, not binding; however, they typically inform the direction of the editorial decision. The Editor may also conduct additional assessment of the manuscript or consult with editorial colleagues on methodological or conceptual points. The statistical editor may be asked to review and add further comments.

The editor then issues one of several possible decisions:

*Reject*: The manuscript is not suitable for publication in its present form, and revisions are unlikely to change that.
*Major revisions*: Substantial clarifications, reanalyses, reframing, or restructuring are needed. Authors will be invited to resubmit, and the revised manuscript may return to the same reviewers.
*Minor revisions*: Changes needed are more limited, typically involving clarification of reporting, refinement of argumentation, or minor stylistic corrections.
*Accept*: Rarely issued without prior revision, indicating that the manuscript meets the standards for publication.

## Stage 6. Revision and resubmission

If revisions are requested, authors are encouraged to respond to reviewer and editorial comments. A well-structured response letter that clearly delineates how each point has been addressed helps facilitate efficient reassessment. A table is particularly helpful. In some cases, clarifying the rationale for maintaining an original interpretation is appropriate, provided this is done transparently and supported by evidence. We don’t expect you (or us!) to agree with everything a reviewer says.^2^

Revised manuscripts undergoing major revisions are commonly re-evaluated by their original reviewers. This step allows reviewers to determine whether their concerns have been adequately addressed. This stage can take a flexible length of time depending on how quickly authors submit revisions, although reviewers are still asked to submit their reviews within 3 weeks.

## Stage 7. Final decision and beyond

When the manuscript is accepted, it moves into production. The paper undergoes copyediting, typesetting, and proofreading. Authors receive proofs for review within about 3 weeks and are responsible for confirming the accuracy of the final version. The article is then published online around 2 weeks later and subsequently assigned to the next available journal issue.

## Conclusions

We hope the editorial and peer review processes are collaborative endeavours which foster rigour, clarity, and relevance in palliative care scholarship. While the process can be lengthy, each step serves an important function in ensuring that Palliative Medicine publishes robust work that advances knowledge, informs clinical practice, strengthens policy, and supports the global palliative care community. Transparency about this process is essential for supporting authors, valuing reviewer expertise, and maintaining the journal’s commitment to high scholarly standards. By providing this overview, we hope to demystify the journey a manuscript takes through Palliative Medicine and to acknowledge the essential contributions of all who participate in it.
